# The effect of sleep apnoea related pathways on longitudinal changes in the brain

**DOI:** 10.1002/alz.090288

**Published:** 2025-01-09

**Authors:** Antoine Weihs, Stefan Frenzel, Ralf Ewert, Beate Stubbe, Thomas Penzel, Ingo Fietze, Robin Bülow, Henry Voelzke, Hans J. Grabe

**Affiliations:** ^1^ University Medicine Greifswald, Greifswald Germany; ^2^ German Centre for Neurodegenerative Diseases (DZNE), Greifswald Germany; ^3^ University Hospital Charité, Berlin Germany; ^4^ The Fourth People's hospital of Guangyuan, Guangyuan City China; ^5^ German Centre for Cardiovascular Research (DZHK), Greifswald Germany

## Abstract

**Background:**

Sleep apnoea is gaining attention as a modifiable risk factor for neurodegenerative disorders, with recent cross‐sectional studies observing associations with advanced brain ageing and increased white matter hyperintensity (WMH) burden. Building upon these findings, we investigate the effects of sleep apnoea on longitudinal changes in brain structural measures using lab‐based polysomnography and 7‐year follow‐up MRI measurements from the general population‐based Study of Health in Pomerania (SHIP).

**Method:**

Ordinary least square regression was used. Brain measures (brain age, total brain volume (TBV), mean cortical thickness and WMH at follow‐up) were chosen as outcomes and sleep apnoea metrics (apnoea hypopnea index (AHI), oxygen desaturation index (ODI), arousal index (ARI) and mean oxygen saturation (meanO2) during sleep) as exposures. All models were adjusted for the corresponding baseline MRI metric, follow‐up time, age*sex, intracranial volume and total sleep time (TST). The exposure and all numerical covariates were modelled non‐linearly using restricted cubic splines. AHI, ODI, TST and meanO2 were manually scored according to American Academy of Sleep Medicine standards. ARI was automatically assessed using Sleepware (Phillips, Amsterdam, Netherlands). Structural MRI measurements were segmented using T1‐weighted images in FreeSurfer v7.2. WMH were segmented with the lesion growth algorithm implemented in the LST toolbox v3.0.0 using T1‐weighted and FLAIR sequences. Additionally, brain age scores were generated to represent age‐related structural brain changes.

**Result:**

N=387 subjects (47.3% female, baseline age 50.7±12.4 years) with baseline and follow‐up MRI (mean follow‐up time: 7.4±0.6 years) and baseline polysomnography measurements were included. No longitudinal effect was identified when investigating sleep apnoea severity (AHI). Looking at apnoea‐related oxygen desaturation subprocesses, only an inverse relationship was observed between meanO2 and TBV. Exploring sleep fragmentation, a positive effect was observed between ARI and brain age and an inverse effect between ARI and TBV and mean cortical thickness. These effects remained stable when additionally accounting for metabolic, socioeconomic or lifestyle factors.

**Conclusion:**

Using longitudinal MRI and lab‐based polysomnography measurements, we found sleep fragmentation to be associated with brain atrophy after 7 years. While further studies are needed, this indicates that sleep fragmentation might be an interesting target to slow the onset of neurodegenerative disorders.

